# Predictors of incident diabetes in two populations: framingham heart study and hispanic community health study / study of latinos

**DOI:** 10.1186/s12889-022-13463-8

**Published:** 2022-05-26

**Authors:** Robert C. Kaplan, Rebecca J. Song, Juan Lin, Vanessa Xanthakis, Simin Hua, Ariel Chernofsky, Kelly R. Evenson, Maura E. Walker, Carmen Cuthbertson, Joanne M. Murabito, Christina Cordero, Martha Daviglus, Krista M. Perreira, Marc Gellman, Daniela Sotres-Alvarez, Ramachandran S. Vasan, Xiaonan Xue, Nicole L. Spartano, Yasmin Mossavar-Rahmani

**Affiliations:** 1 Department of Epidemiology and Population Health, Albert Einstein College of Medicine, 1300 Morris Park Avenue. Belfer building, Room 1315, Bronx, NY 10461 USA; 2 Public Health Sciences Division, Fred Hutchinson Cancer Research Center, Seattle, WA USA; 3 Department of Epidemiology, Boston University School of Public Health, Boston, MA USA; 4 Department of Medicine, Boston University School of Medicine, Boston, MA USA; 5 Department of Biostatistics, Boston University, Boston, MA USA; 6 Department of Epidemiology Gillings School of Global Public Health, University of North Carolina at Chapel Hill, Chapel Hill, NC USA; 7 Department of Health Sciences, Boston University College of Health & Rehabilitation Sciences, Boston, MA USA; 8 Department of Epidemiology, University of North Carolina at Chapel Hill, Chapel Hill, NC USA; 9 Department of Psychology, Don Soffer Clinical Research Center, University of Miami, Miami, FL USA; 10 Institute for Minority Health Research, University of Illinois at Chicago, Chicago, IL USA; 11 Department of Social Medicine, University of North Carolina School of Medicine, Chapel Hill, NC USA; 12 Department of Psychology, University of Miami, Miami, FL USA; 13 Department of Biostatistics, University of North Carolina at Chapel Hill, Chapel Hill, NC USA

**Keywords:** diabetes, Hispanic, Latino, risk factors, occupation, epidemiology, physical activity

## Abstract

**Background:**

Non-genetic factors contribute to differences in diabetes risk across race/ethnic and socioeconomic groups, which raises the question of whether effects of predictors of diabetes are similar across populations. We studied diabetes incidence in the primarily non-Hispanic White Framingham Heart Study (FHS, *N* = 4066) and the urban, largely immigrant Hispanic Community Health Study/Study of Latinos (HCHS/SOL, *N* = 6891) Please check if the affiliations are captured and presented correctly.

**Methods:**

Clinical, behavioral, and socioeconomic characteristics were collected at in-person examinations followed by seven-day accelerometry. Among individuals without diabetes, Cox proportional hazards regression models (both age- and sex-adjusted, and then multivariable-adjusted for all candidate predictors) identified predictors of incident diabetes over a decade of follow-up, defined using clinical history or laboratory assessments.

**Results:**

Four independent predictors were shared between FHS and HCHS/SOL. In each cohort, the multivariable-adjusted hazard of diabetes increased by approximately 50% for every ten-year increment of age and every five-unit increment of body mass index (BMI), and was 50–70% higher among hypertensive than among non-hypertensive individuals (all *P* < 0.01). Compared with full-time employment status, the multivariable-adjusted hazard ratio (HR) and 95% confidence interval (CI) for part-time employment was 0.61 (0.37,1.00) in FHS and 0.62 (0.41,0.95) in HCHS/SOL. Moderate-to-vigorous physical activity (MVPA) was an additional predictor in common observed in age- and sex-adjusted models, which did not persist after adjustment for other covariates (compared with MVPA ≤ 5 min/day, HR for MVPA level ≥ 30 min/day was 0.48 [0.31,0.74] in FHS and 0.74 [0.56,0.97] in HCHS/SOL). Additional predictors found in sex- and age-adjusted analyses among the FHS participants included male gender and lower education, but these predictors were not found to be independent of others in multivariable adjusted models, nor were they associated with diabetes risk among HCHS/SOL adults.

**Conclusions:**

The same four independent predictors – age, body mass index, hypertension and employment status – were associated with diabetes risk across two disparate US populations. While the reason for elevated diabetes risk in full-time workers is unclear, the findings suggest that diabetes may be part of the work-related burden of disease. Our findings also support prior evidence that differences by gender and socioeconomic position in diabetes risk are not universally present across populations.

**Supplementary Information:**

The online version contains supplementary material available at 10.1186/s12889-022-13463-8.

## Introduction

The diabetes epidemic is growing across the US and globally [1], although the disease burden is concentrated in certain race/ethnicity populations. Clinical guidelines recommend more aggressive approaches for diabetes surveillance among Hispanic or Latino, Black/African American, and other race/ethnic groups than among non-Hispanic whites demonstrating the public health significance of population disparities in diabetes risk [2].

While diabetes risk is affected by genetic predisposition, it is well known that lifestyle elements including diet, physical activity, and sleep remain important predictors among those at both low and high genetic risk [3]. Moreover, social and lifestyle factors might explain differences in diabetes risk across race/ethnic, cultural and socioeconomic groups, raising an important question about whether the contribution of specific characteristics to diabetes propensity are similar across populations. For instance, studies have identified excess adiposity and low physical activity as predictors of diabetes risk that may have similar magnitude of associations across US race/ethnic groups [4–14]. Several of the previous studies enrolled modest numbers of participants outside of non-Hispanic white groups [14], evaluated a limited age range [6, 7, 13], focused exclusively on women [6–8], or had other eligibility restrictions that might have contributed to non-representative samples [8, 10].

Our prospective analyses compared the effects of predictors of incident diabetes among over 10,000 individuals drawn from different well-defined communities. The Framingham Heart Study (FHS) is a cohort of primarily non-Hispanic white adults recruited from the moderate-sized town of Framingham, Massachusetts (population ~ 74,000, density = 2,971/square mile) and its vicinity [15]. The Hispanic Community Health Study / Study of Latinos (HCHS/SOL) recruited an area-based sample of Hispanic / Latino residents of four densely-populated US cities. These two studies both used area-based sampling and recruitment methods, have a similar age distribution, and were conducted using similar epidemiological methods, yet each captures a distinct segment of the US population. Thus we compared the important sociodemographic, clinical, and behavioral predictors of incident diabetes across two disparate populations through parallel analyses among the FHS cohort of highly educated, predominantly non-Hispanic white adults and HCHS/SOL’s largely foreign-born, low socioeconomic position (SEP) population living in urban Hispanic enclaves.

## Methods

### Study populations

Multiple cohorts from the FHS that had accelerometry measurements were included. The FHS Offspring cohort began enrollment in 1971, targeting the children of the original FHS cohort and the children’s spouses [16]. In 1994, FHS enrolled the Omni-1 cohort members, consisting of Framingham residents who self-identified as members of a minority group [17, 18]. In 2002, the children of the Offspring cohort (Third Generation), spouses of the Offspring who were not previously enrolled in the study (New Offspring Spouses, NOS), and another minority Omni-2 cohort of Framingham residents were enrolled [19]. All participants from these FHS cohorts are invited to participate in examinations approximately every 4 years at which time data regarding demographic information, medications, medical and family history, clinical measurements, and health behaviors are collected. In the interim between examinations, participants are contacted via phone and email for their annual health history interviews to track medical history. Vital statistics data are also ascertained from physician office records and death certificates. Here we included participants who attended the ninth examination cycle of the Offspring cohort, the fourth examination cycle of the Omni-1 group (both during 2011–2014) or the second examination cycle of the Third Generation, NOS and Omni-2 cohorts (2008–2011). All participants provided written informed consent, and the Institutional Review Board at Boston University Medical Center approved the study protocols. All methods were performed in accordance with the relevant guidelines and regulations.

HCHS/SOL is a longitudinal cohort study initiated in 2008 among a 16,415-person sample of Hispanic or Latino adults aged 18 to 74 years. HCHS/SOL participants, four-fifths of whom were born outside the 50 US states, were a population based (area) sample of Bronx, NY, Chicago, IL, Miami, FL, and San Diego, CA. Relative to the communities that were sampled, the HCHS/SOL recruitment strategy was designed to oversample individuals above 45 years of age, in order to better study diseases affecting the middle-aged and older population. At baseline, HCHS/SOL used interviews in the language of participants’ preference to ascertain demographic data, education, income, currently held occupation, medical history, medications and health behaviors. Standardized clinical measures included height, weight, seated blood pressures (BP) and overnight fasting venous blood collection to capture metabolic laboratory tests. Key variables were updated by annual telephone interviews and at a six year follow up visit. Follow-up for episodes of hospitalization or emergency department use and mortality was based upon annual contact attempts, next-of-kin reports and search of vital statistics records. All participants provided informed consent, and human subjects oversight was conducted by the four field center institutions and the HCHS/SOL coordinating center.

The flow chart in Supplemental Fig. 1 describes participant selection and inclusion criteria.

### Definition of incident diabetes

Study baseline for each participant was defined according to their research study visit date during the 2008–2011 examination cycle for the HCHS/SOL cohort, the 2008–2011 examination cycle for the FHS Third Generation, Omni-2, and NOS cohorts, or the 2011–2014 examination cycle for the FHS Offspring and Omni-1 cohorts (Supplemental Table 1). To exclude individuals with prevalent diabetes at baseline, both cohorts used self-reported clinical diagnosis and treatment information as well as laboratory measurements performed as part of the study including fasting blood glucose ≥ 126 mg/dL and hemoglobin A1c ≥ 6.5%. Incident diabetes was defined by either 1) a physician diagnosis of diabetes and the use of diabetes medications, based on self-reported information obtained at an annual telephone follow-up or an in-person cohort examination, or 2) measured glycemic traits at a follow-up study examination, including the American Diabetes Association (ADA) criteria of fasting glucose ≥ 126 mg/dl (both cohorts) or hemoglobin A1c ≥ 6.5% (HCHS/SOL cohort only). HCHS/SOL and FHS used a hexokinase enzymatic method for plasma glucose (Roche Diagnostics Corporation, Indianapolis, IN). For measurement of HbA1c, HCHS/SOL used liquid chromatography in EDTA-anticoagulated whole blood (Tosoh G7 analyzer, Tosoh Bioscience, San Francisco, CA) and FHS used a Roche Cobas 501 or Roche Hitachi 911 analyzer (Roche Diagnostics, Indianapolis, IN).

### Covariate Definitions

Covariates including medical history, medication use, health related behaviors, socioeconomic variables, and anthropometric variables were obtained from either standardized self-reported questionnaires or examination procedures performed at an in-person study examination, as detailed in Supplemental Table 1. In addition, physical activity assessment was performed with similar protocols in FHS and HCHS/SOL using an Actical version B-1 (model 198–0200-03; Respironics Co., Bend, OR) accelerometer, positioned above the iliac crest and worn for seven days. To ensure reliable estimates for physical activity and sedentary time, only participants who adhered to the accelerometer protocol, defined as at least three days of >  = 10 h of wear each day, were included. Because total sedentary time depends on wear time, we standardized total sedentary time to reflect 16 h of wear time per day using the residuals obtained from regressing sedentary time on wear time. As a result, total sedentary time was calculated as an average across days with wear-time that met the bar for adherence and expressed as the mean predicted sedentary time given a wear time of 16 h per day.

### Statistical analyses

Within-cohort analyses used Cox proportional hazards regression to examine the association between baseline levels of potential predictor variables and incident diabetes expressed as hazard ratios (HR) and their 95% confidence intervals. Time to event was defined according to days since the study baseline visit. The date of an incident diabetes event was defined at the time of the first self-report of diabetes diagnosis during an annual follow-up interview or an in-person follow-up examination. In addition, for the HCHS/SOL and FHS Third Generation cohorts which had a repeat examination during the follow-up period, the date of the subsequent follow-up clinical examination was used, in the case where incident diabetes was detected according to levels of measured fasting glucose or hemoglobin A1c (HCHS/SOL examination cycle 2 during 2014–2017 and FHS Third Generation examination cycle 3 during 2016–2019).

Variables considered as potential predictors of incident diabetes included age, sex, education, marital status, employment, smoking, alcohol use, body mass index (BMI) (per unit, kg/m^2^), the Alternative Healthy Eating Index (AHEI)-2010 score (per unit, range 0–110), moderate-to-vigorous physical activity (MVPA), sedentary time, average accelerometer counts per minute as a measure of total volume of physical activity, hypertension defined by use of antihypertensive medications or measured BP above 140/90 mmHg, and use of lipid-lowering medication and aspirin. HCHS/SOL analyses additionally incorporated adjustment for field center, Hispanic/Latino background, and health insurance status (FHS did not because the cohort was nearly universally insured). For descriptive purposes, we used a previously published typology to assign typical metabolic equivalent values (METs) to self-reported job titles, in order to describe the degree of exertion associated with each person’s field of employment [20–23].

In our initial models to identify predictors of incident diabetes, we adjusted for age and sex only. Correlations between sedentary time and MVPA were moderate (*r* = -0.41 in FHS and *r* = -0.49 in HCHS/SOL), thus all models used to examine the association of MVPA and sedentary time with risk of diabetes included both of these variables together in the model (correlations among other covariates were low-to-moderate). Finally, all candidate predictor variables, regardless of their significance in age and sex adjusted models, were included together in multivariable models in order to identify those that were independent predictors of incident diabetes. The exception to this was the accelerometry data; total counts per minute was the only accelerometry metric included in our final multivariable models. Alternate approaches where we included adjustment for MVPA or sedentary time rather than total counts per minute as independent variables did not change our conclusions regarding predictors of incident diabetes (data not shown). Statistically significant independent variables were identified by the *P* < 0.05 criterion. We estimated the C-statistic for the fully adjusted models as a metric of model fit.

Missing covariates were handled using complete case approach, and all independent variables had 6% or fewer missing. Stratification, clustering and survey sampling weights were used in HCHS/SOL analyses to account for its complex sampling design. A sensitivity analysis was conducted only among FHS participants who were non-Hispanic white. All HCHS/SOL participants completed the follow-up examination, and only 26 of the FHS participants lacked follow-up information, so loss-to-follow-up was considered to be modest. Visual examination of plots of Schoenfeld residuals was used to confirm that hazards were proportional over follow-up time.

Statistical analyses were conducted using R.3.6.3 (R Project for Statistical Computing, Geneva) and SAS version 9.4 (SAS Institute Inc, Cary, NC).

## Results

In both FHS (*N* = 4,066) and HCHS/SOL cohorts (*N* = 6,891), about 40% were men (Table 1). The largest age group was 45 to 54 years old in both cohorts. As compared with FHS participants, HCHS/SOL adults had worse self-reported overall health, worse AHEI-2010 diet quality score, and a higher prevalence of overweight, obesity and smoking. Hypertension and use of preventive medications (lipid-lowering, aspirin) were more common among FHS participants than among HCHS/SOL participants. Only half of HCHS/SOL adults reported having health insurance, while almost all FHS participants were insured and had made a healthcare visit in the year preceding their baseline FHS examination. Education and income were higher in FHS compared with HCHS/SOL. Employment characteristics differed markedly between cohorts with FHS being mostly employed (57.2% full-time and 16.0% part-time, versus 10.6% unemployed) whereas in HCHS/SOL the number of unemployed nearly equaled the number of full-time workers (36.2% and 38.1%, respectively). Over 60% of FHS participants had an annual household income over $50,000 suggesting they were likely to hold relatively high-status jobs. In contrast to FHS participants, only 10.8% of HCHS/SOL adults had an annual household income over $50,000, and more than 40% of HCHS/SOL adults had an annual household income under $20,000 (versus 11.4% of FHS).

**Table 1:** Baseline sample characteristics in FHS and HCHS/SOL

	FHS	HCHS/SOL
No. of participants	4066	6891
Baseline year, median (IQR)	2010 (2009, 2011)	2010 (2009, 2010)
*Demographic characteristics*		
Age in years, mean (SD)	53.9 (13.6)	45.6 (13.0)
Age group, %		
18–34	6.6	21.0
35–44	20.3	19.5
45–54	28.4	33.7
55–64	20.4	20.0
65–74	16.5	5.9
75 +	7.8	0
Sex, % male	43.7	36.6
Race and ethnicity, %		
Non-Hispanic White	90.7	0
Hispanic	3.8	100
Black/African-American	2.0	0
Mixed/other	3.5	0
Employment Status, %		
Retired and not employed	16.2	6.8
Unemployed (nonemployed, nonretired)	10.6	36.2
Part-time	16.0	18.9
Full-time	57.2	38.1
Marital status, %		
Married or living with a partner	73.0	55.9
Divorced or separated	11.8	24.9
Single, never married or widowed	15.2	19.3
Annual family income, %		
< $20,000	11.4	40.9
$20,000–50,000	23.1	40.8
> $50,000	52.4	10.8
Not reported	13.1	7.4
Education, %		
Less than High School	1.2	34.7
High School or GED	15.1	26.0
Greater than high school	83.7	39.3
*Clinical and healthcare*		
Self reported general health, %		
Excellent	25.4	8.9
Very good	49.2	17.4
Good	23.0	49.6
Fair	2.2	21.1
Poor	0.2	3.0
Overweight, %	36.3	40.9
Obese, %	26.6	37.2
Lipid lowering medication, %	21.8	6.7
Hypertension, %	24.8	10.1
Aspirin use, %	24.1	17.7
Health insurance, %	99.2	48.2
Healthcare use, %	94.3	70.7
*Health behavior*		
Current smoking, %	7.4	17.2
Current alcohol use, % yes	82.0	49.4
Alternate Healthy Eating Index-2010, median (IQR)	63.0 (53.7, 72.1)	49.0 (43.8, 54.6)
MPVA in minutes/day, median (IQR)	13.8 (5.5, 26.6)	15.7 (6.5, 31.0)
Light activity in minutes/day, median (IQR)	191.0 (147.1, 242.1)	221.7 (169.6, 289.4)
Total physical activity in minutes/day, median (IQR)	209.8 (163.3, 264.6)	243.0 (184.7, 316.5)
Average counts per minute, median (IQR)	136.9 (94.2, 196.4)	146.7 (101.6, 212.6)
Sedentary minutes/day, median (IQR)	731.3 (684.6, 773.0)	713.8 (645.4,771.3)

*SD* standard deviation, *IQR* interquartile range, *MVPA* moderate to vigorous physical activity

Follow-up continued up to 10.8 years in FHS (median, 8.3 years) and up to 9.6 years in HCHS/SOL (median 5.8 years). At the end of the follow-up period, in FHS we observed 240 incident diabetes cases for a cumulative incidence of 5.9%, while in HCHS/SOL we observed 1,132 incident diabetes cases for a cumulative incidence of 16.4%.

### Predictors of incident diabetes in age and sex adjusted models

Table 2 presents age- and sex- adjusted hazard ratios of diabetes for 14 candidate predictor variables. Four were statistically significant in both FHS and HCHS/SOL cohorts: age (HR per one year increment = 1.04, *P* < 0.01 in both cohorts), BMI (HR per one unit increment = 1.11 in FHS and 1.07 in HCHS/SOL, both *P* < 0.01), hypertension (HR = 2.37 in FHS and 1.71 in HCHS/SOL, both *P* < 0.01), and MVPA level ≥ 30 min/day (versus the reference group of ≤ 5 min/day, HR = 0.48, *P* < 0.01 in FHS and HR = 0.74, *P* = 0.03 in HCHS/SOL). In FHS, total accelerometer counts per minute < 200 was associated with risk of diabetes (versus ≤ 90 counts per minute, HR = 0.56, *P* = 0.01), while the HR for the highest sedentary time category approached statistical significance (HR = 1.49, *P* = 0.06 comparing ≥ 780 min/day versus ≤ 660 min/day). In contrast, neither total counts per minute nor sedentary time was associated with incident diabetes in HCHS/SOL. In FHS, but not HCHS/SOL, we observed additional variables that predicted a higher risk of diabetes in age- and sex-adjusted models, including male sex, full-time employment, divorced or separated marital status, use of lipid-lowering treatment and current smoking.

**Table 2:**
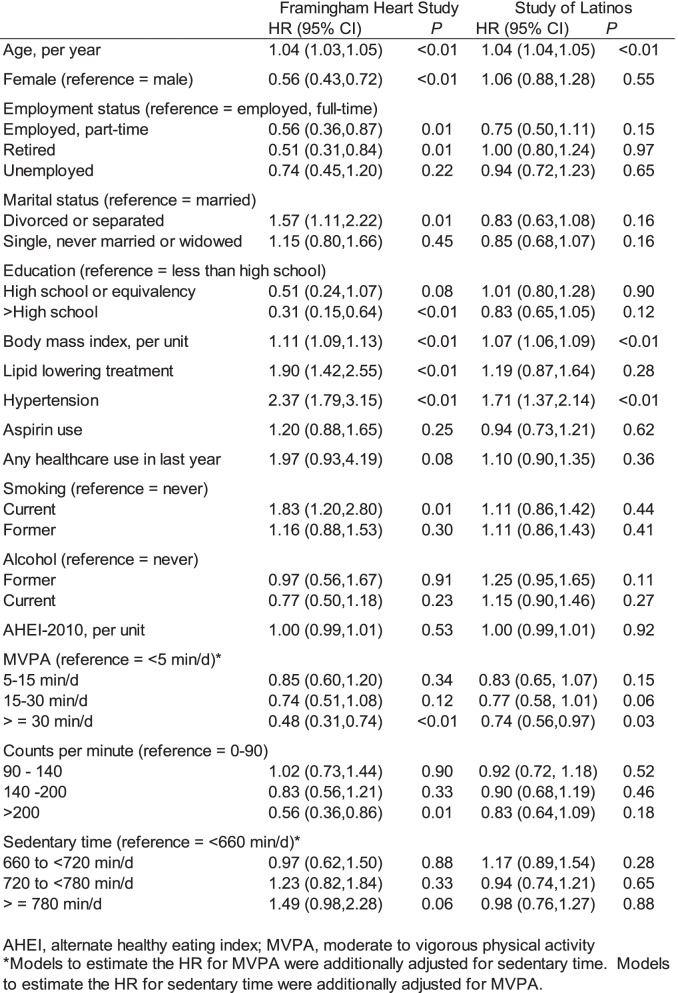
Risk factors for incident diabetes, adjusted for age and sex

### Multivariable analyses of predictors of incident diabetes

Table 3 presents hazard ratios of diabetes when all 14 candidate predictors were included in the model. Consistent with age- and sex-adjusted models, this multivariable analysis revealed that advanced age, higher BMI and hypertension were predictors of incident diabetes in both FHS and HCHS/SOL cohorts (all *P* < 0.01). Effect estimates were consistent with a 50–70% increase in relative hazard of diabetes associated with hypertension. We observed approximately a 50% increase in hazards for every ten years of age (HR = 1.04, therefore HR^10^ = 1.48 in FHS and HR = 1.05, HR^10^ = 1.63 in HCHS/SOL) and every five units of BMI (HR = 1.09, HR^5^ = 1.54 in FHS, and HR = 1.07, HR^5^ = 1.40 in HCHS/SOL).

**Table 3:**
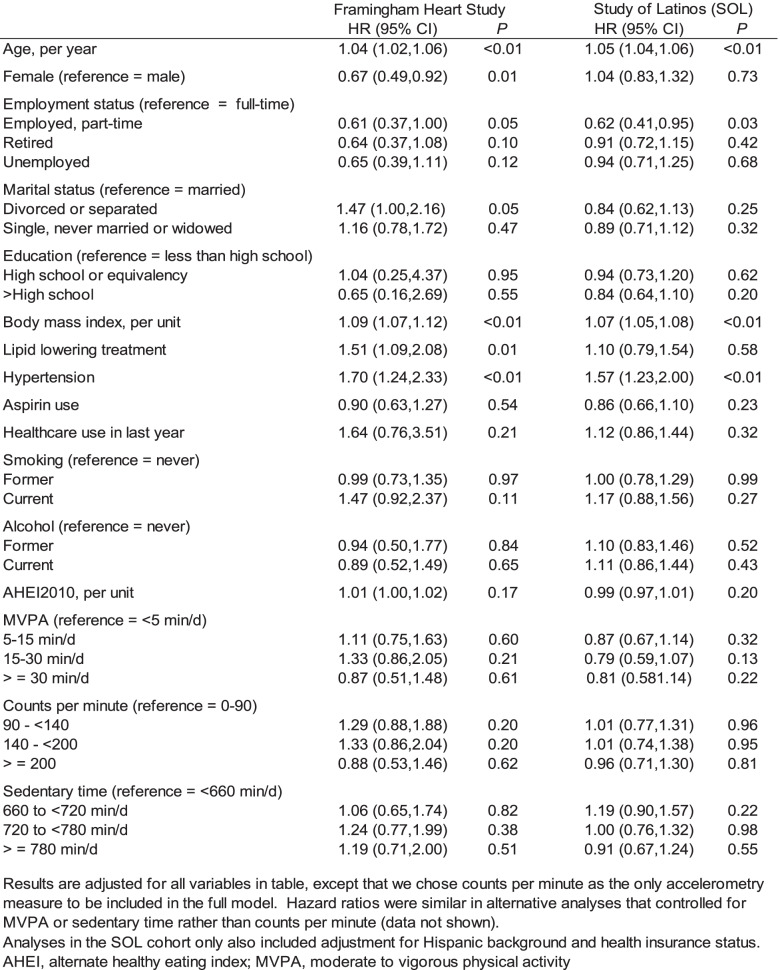
Multivariable analyses of risk factors for incident diabetes

A fourth independent predictor that appeared to persist in multivariable analyses, with nearly identical effect size in each cohort, was full-time employment status. The hazard ratio for part-time versus full-time employment was 0.61 (*P* = 0.05) in FHS and 0.62 (*P* = 0.03) in HCHS/SOL. In considering potential mediators of this association, we noticed that the nature of employment differed between the FHS and HCHS/SOL cohorts. Over half of employed individuals in HCHS/SOL (54%) had jobs associated with moderate-to-high levels of physical activity (METs > 3). Only 7% of employed FHS participants worked in highly physical jobs, being more likely than HCHS/SOL adults to hold sedentary jobs (43%, with typical METs < 2, versus 15% in HCHS/SOL). In both cohorts, MVPA and accelerometry counts per minute were highest in full-time employees, lowest in retirees, and intermediate in part-time employees (Supplemental Table 2 and Supplemental Table 3). Sedentary time was highest in retirees, and lowest in the employed, especially in those with jobs typically associated with high METs.

Other statistically significant predictors of higher diabetes that were observed in FHS but not in HCHS/SOL (Table 3) included being male, divorced or separated, and using lipid-lowering medication.

The C-statistics for the overall prediction of diabetes risk was observed to be 0.767 in FHS and 0.704 in HCHS/SOL.

Analyses in FHS were substantially similar when limited to the > 90% of the population of non-Hispanic white background (Supplemental Table 4).

## Discussion

The present investigation examined predictors of incident diabetes in two distinct populations, one comprising mostly non-US-born Hispanic/Latino city dwellers with low education and income (HCHS/SOL), and the other representing a higher SEP, primarily white non-Hispanic population (FHS). We drew similar conclusions from each cohort regarding the leading independent risk predictors for diabetes. Despite a much higher diabetes incidence among our Latino cohort, the same three clinical variables – age, BMI and hypertension – were predictors in common having nearly identical relative hazards of incident diabetes in HCHS/SOL and FHS. These predictors of incident diabetes were independent of each other, achieved a high level of statistical significance, and persisted after adjustment for an array of clinical, behavioral and socioeconomic variables.

Full-time employees had an elevated risk of diabetes in comparison with those employed part-time, with a HR for part-time versus full-time employees of ~ 0.6 in each cohort. The association between employment and diabetes risk may not be widely recognized, but a large meta-analysis estimated with high precision a risk ratio for diabetes of 0.86 (95% CI 0.78, 0.95) comparing part-time workers (less than 35 h per week) with those working 35–45 h [24]. Psychological aspects of work that have been related to diabetes risk including job strain [25] were not addressed by our study. Chemical exposures in the workplace may also pose a risk of diabetes [26], although in HCHS/SOL we previously observed no association between exposure to solvents, metals or pesticides and fasting glucose levels [27]. HCHS/SOL data have linked longer working hours with obesity [22], yet full-time employment remained associated with elevated diabetes risk after adjustment for BMI as well as behaviors influenced by work (including physical activity and diet quality).

High levels of MVPA (equal to or exceeding 30 min per day) had a statistically significant association with reduced risk of diabetes. In FHS, but not in HCHS/SOL, low sedentary time and high total physical activity were also associated with a lower risk of diabetes. However, after adjustment for variables such as employment, hypertension and BMI, in neither cohort were accelerometry-derived measures of physical activity independently associated with risk of diabetes. This may be considered a form of over-adjustment, since for example prevention of obesity by an active lifestyle could account for some of the benefit of physical activity. While a large number of studies showed an association between greater self-reported physical activity and lower risk of incident diabetes [28], more recent studies using accelerometry or pedometry have sometimes [29] but not consistently [30] reported that this association exists.

The underlying hazard of diabetes increased by approximately 50% for every ten years increment of age and every five-unit increment of BMI. This confirms prior evidence that at a population-wide level, older age and excess adiposity substantially determine an individual’s risk of diabetes [6]. The association with BMI persisted after adjustment for obesity-driven factors including hypertension and low physical activity, such that the adjusted analyses might underestimate the true risks of diabetes associated with excess adiposity. The low prevalence of individuals with the recommended levels of BMI below 25 kg/m^2^ in both HCHS/SOL (21.9%) and FHS (37.1%) reminds us of the primary importance of excess adiposity as a modifiable target for preventing diabetes across a wide range of populations. Hypertension is another known predictor of diabetes risk that was confirmed across the two populations in our study. Prior research suggests this association may be related to the presence of hypertension per se rather than to side effects of antihypertensive medications [31]. Thus, all hypertensive patients might benefit from close monitoring for evidence of diabetes.

Other clinical predictors of diabetes risk were identified in age-and sex-adjusted analyses (but not multivariable-adjusted models), although these were present only in the FHS cohort and not the HCHS/SOL cohort, namely lipid-lowering treatment and current smoking. Infrequent use of lipid-lowering treatment in the HCHS/SOL population may have affected our power to detect this association. The small risk of diabetes associated with use of statins is already recognized [32] and that does not negate the powerful cardiovascular benefits of lipid-lowering treatments. The lack of association between smoking and incident diabetes in HCHS/SOL may be explained by the relatively light intensity smoking habits of our Latino population [33].

In the predominantly non-Hispanic white FHS cohort, greater educational attainment mitigated the risk of incident diabetes after adjustment for age and sex. This association did not persist in multivariable models, but since the adjustment variables included potential mediators (such as obesity and hypertension), the results could be interpreted as confirmation that individuals with a low SEP are a high-risk group. This observation is consistent with a meta-analysis of 23 studies which concluded that the lowest categories of education and income were associated with a ~ 40% increase in relative risk of diabetes relative to the highest categories [34]. Among HCHS/SOL Hispanic/Latino adults, diabetes risk did not vary significantly by level of education, which may be related to the fact that their education may have been obtained outside of the US. Other studies also suggest a different relationship between SEP and health among immigrants as compared with the overall US population. For example, it is known from prior studies such as the San Antonio Heart Study that rising SEP among US Latinos can be associated with worsening rather than improvements in metabolic health [35]. Additional factors identified in FHS only, but not in HCHS/SOL, were being male, divorced or single. This points to the potential importance of ethnic sociocultural differences such as greater social support among the Latino population which may protect against diabetes risk [36]. Finally, our study’s design was best suited to identify predictors that were shared across populations, which does not negate the importance of structural and interpersonal dimensions of disadvantage which are fundamental to the high burden of diabetes among Latinos [37].

Study limitations include some differences in the design of the two cohort studies, such as the schedule of follow-up contacts, the recruitment approaches and the community settings. Differences in methodology may strengthen our conclusions to some extent, showing that associations between predictors and incidence of diabetes are robust and generalizable, despite distinct patterns of confounding and selection biases in each cohort. For example, it was striking that the number of working hours had a similar relationship with diabetes risk in each of our cohorts, despite differences between FHS and HCHS/SOL in the prevailing types of employment (being mainly sedentary jobs in the former group, and active jobs in the latter). However, further research will be needed to gain a more nuanced understanding of potential occupational health disparities and the interaction of work with other influences on health such as race/ethnicity, immigration status, gender and socioeconomic position [38]. Unmeasured confounding is another possible limitation, and while diet is an important aspect of diabetes prevention we did not focus on nutritional influences on diabetes in the present investigation.

## Conclusion

We identified age, BMI, hypertension and full-time employment as four independent and replicable predictors of the risk of diabetes in two large community-based samples. Our multi-cohort approach allowed us to find generalizable predictors that may be targeted in universal approaches to prevention [39]. The focus on universal predictors offers the benefit of simplicity and avoids the difficulties associated with defining the membership of a “population”. Finally, our findings add to the recent data that have raised concerns about the work-related burden of disease worldwide [40].

## Supplementary Information

**Additional file 1: Supplemental Figure 1.** Flow diagram of participant inclusion and exclusion.

**Additional file 2: Supplemental Table 1.** Variable definitions in Framingham Heart Study and Hispanic Community Health Study/Study of Latinos. **Supplemental Table 2.** Demographic and physical activity characteristics by employment status, Framingham Heart Study. **Supplemental Table 3.** Demographic and physical activity characteristics by employment status, Hispanic Community Health Study / Study of Latinos. **Supplemental Table 4.** Multivariable analyses of risk factors for incident diabetes, among non Hispanic whites from Framingham Heart Study.

## Data Availability

The datasets generated and/or analyzed during the current study are not publicly available due to policies of the funding agency, but are available from the corresponding author on reasonable request.
