# Supplementary material for: Predictors of incident diabetes in two populations: framingham heart study and hispanic community health study / study of latinos
Source: BMC Public Health. 2022 May 26;22:1053. doi: 10.1186/s12889-022-13463-8 (PMC9137165; doi:10.1186/s12889-022-13463-8)
Supplement: Supplementary file 1 — Additional file 1: Supplemental Figure 1. Flow diagram of participant inclusion and exclusion. [file 12889_2022_13463_MOESM1_ESM.pdf]

Supplemental Figure 1. Flow diagram of participant inclusion and exclusion

Hispanic Community Health Study/Study of Latinos cohort (HCHS/SOL)

16,415	Enrolled in the study	→	4,792	Excluded, no second examination
11,623	Completed second examination	→	2,546	Excluded, prevalent or missing diabetes status
9,077	At risk for incident diabetes	→	428	Excluded, prevalent coronary heart disease or stroke
8,649	Free of vascular disease	→	1,758	Excluded, participants not adherent to accelerometry
6,891	Completed accelerometry			
6,891	Final HCHS/SOL sample			
42%	Percent of cohort included			

Framingham Heart Study Gen 2, Gen 3, Omni 1 and Omni 2 cohorts

10,239	Enrolled in the study	→	5,530	Excluded, participants not adherent to accelerometry
4,709	Completed accelerometry	→	414	Excluded, prevalent or missing diabetes status
4,295	At risk for incident diabetes	→	196	Excluded, prevalent coronary heart disease or stroke
4,099	Free of vascular disease	→	33	Excluded, missing data on pre-diabetes
4,066	Data available to assess pre-diabetes status			
4,066	Final FHS sample			
40%	Percent of cohort included			
