# Supplementary material for: Predictors of incident diabetes in two populations: framingham heart study and hispanic community health study / study of latinos
Source: BMC Public Health. 2022 May 26;22:1053. doi: 10.1186/s12889-022-13463-8 (PMC9137165; doi:10.1186/s12889-022-13463-8)
Supplement: Supplementary file 2 — Additional file 2: Supplemental Table 1. Variable definitions in Framingham Heart Study and Hispanic Community Health Study/Study of Latinos. Supplemental Table 2. Demographic and physical activity characteristics by employment status, Framingham Heart Study. Supplemental Table 3. Demographic and physical activity characteristics by employment status, Hispanic Community Health Study / Study of Latinos. Supplemental Table 4. Multivariable analyses of risk factors for incident diabetes, among non Hispanic whites from Framingham Heart Study. [file 12889_2022_13463_MOESM2_ESM.docx]

Supplemental tables

**Supplemental Table 1. Variable definitions in Framingham Heart Study and Hispanic Community Health Study/ Study of Latinos**

**Supplemental Table 2. Demographic and physical activity characteristics by employment status, Framingham Heart Study**

**Supplemental Table 3. Demographic and physical activity characteristics by employment status, Hispanic Community Health Study / Study of Latinos**

**Supplemental Table 4. Multivariable analyses of risk factors for incident diabetes, among non Hispanic whites from Framingham Heart Study**

| **Supplemental table 1. Variable definitions in Framingham Heart Study and Hispanic Community Health Study / Study of Latinos** | | |  |
| --- | --- | --- | --- |
|  | **Framingham Heart Study Third Generation/New Offspring Spouses/Omni-2** | **Framingham Heart Study Second Generation/Omni-1** | **Hispanic Community Health Study / Study of Latinos (HCHS/SOL)** |
|  |  |  |  |
| Year of recruitment | 2002 | Offspring, 1971 and Omni-1, 1994 | 2008-2011 |
| Baseline examination for incident diabetes analyses | 2008-2011 (Exam 2) | 2011-2014 (Exam 9) | 2008-2011 (Exam 1) |
| Year of follow up examination | 2016-2019 (Exam 3) | None | 2014-2017 (Exam 2) |
| Frequency of telephone follow up | Annual | | |
| Field center location | One site, Framingham MA | One site, Framingham MA | Four sites, Bronx NY; Chicago IL; Miami FL; San Diego, CA |
| Sex at birth | Self report, baseline | | |
| Education, income, occupation, martial status, race/ethnicity | Self report, baseline | | |
| Health insurance | Not used | Not used | Self report, baseline |
| Country of birth | Not used | Not used | Self report, baseline |
| Weight, height, blood pressure | Measured, baseline | | |
| Body mass index | Calculated from height and weight as kg/m^2^. Overweight, 25-30. Obese, 30 and above. | | |
| Medical history, healthcare use, smoking, alcohol use, perceived general health | Self report, baseline | | |
| Hypertension | Measured systolic blood pressure ≥140 mmHg, diastolic blood pressure ≥ 90 mmHg, or use of antihypertensive medications. | | |
| Medication use | Self-report, baseline | Self-report, baseline | Inventory method, baseline |
| Diet: Raw data collection | Food frequency questionnaire, baseline. | Food frequency questionnaire, baseline. | Two 24 hour dietary recalls, baseline plus food propensity questionnaire at 1^st^ year of follow-up. |
| Diet: Analytic variables | Alternate Healthy Eating Index-2010 calculated using the method of S E Chiuve et al. (2012) “Alternative Dietary Indices Both Strongly Predict Risk of Chronic Disease”; J. Nutr. 142: 1009 – 1018. | | |
| Physical activity: Raw data collection | 7 day accelerometry (Actical), baseline, worn 24 hours per day except for bathing and swimming. | 7 day accelerometry (Actical), baseline, worn during waking hours except for bathing and swimming. | 7 day accelerometry (Actical), baseline, worn during waking hours except for bathing and swimming. |
| Physical activity: Analytic variables | Classified as sedentary time (counts/minute <100), light activity (counts/minute 100 -1535), moderate activity (counts/minute 1535-3960), and vigorous activity (counts/minute >3960). Sedentary time, moderate-to-vigorous physical activity, and average counts/minute were analyzed as approximate quartiles. Nonwear time was removed based on the method of Choi, L., et al., Validation of accelerometer wear and nonwear time classification algorithm. Med Sci Sports Exerc, 2011. 43(2): p. 357-64. Sedentary time was standardized to a 16 hour wear period. | | |
| Diabetes: Baseline (exclusion) | Either 1) a physician diagnosis of diabetes and the use of diabetes medications, or 2) measured glycemic traits, including the American Diabetes Association (ADA) criteria of fasting glucose ≥126 mg/dl or hemoglobin A1c ≥ 6.5% | | |
| Diabetes: Follow-up (incident) | Either 1) a physician diagnosis of diabetes and the use of diabetes medications, based on self-reported information obtained at an annual telephone follow-up or an in-person cohort examination, or 2) measured fasting glucose ≥126 mg/dl at a follow-up study examination | Physician diagnosis of diabetes and the use of diabetes medications, based on self-reported information obtained at an annual telephone follow-up or an in-person cohort examination | Either 1) a physician diagnosis of diabetes and the use of diabetes medications, based on self-reported information obtained at an annual telephone follow-up or an in-person cohort examination, or 2) measured fasting glucose ≥126 mg/dl or hemoglobin A1c ≥ 6.5% at a follow-up study examination |
